# The use of pegylated liposomal doxorubicin in metastatic soft tissue sarcoma

**DOI:** 10.2340/1651-226X.2025.43263

**Published:** 2025-04-23

**Authors:** Trang Pham, Hanne Krogh Rose, Philip Rossen, Ninna Aggerholm-Pedersen

**Affiliations:** a Department of Oncology, Aarhus University Hospital, Aarhus, Denmark; b Department of Clinical Medicine, Aarhus University, Aarhus, Denmark

**Keywords:** Sarcoma, treatment, survival, rare cancer

## Abstract

**Background:**

Soft tissue sarcoma (STS) is a heterogeneous group of rare malignancies with limited response to conventional chemotherapy. Among these, epithelioid haemangioendothelioma (EHE) and angiosarcoma represent rare vascular sarcomas with distinct clinical behaviours, challenging treatment approaches, and poor prognoses. Doxorubicin remains the standard first-line therapy for metastatic STS, but its use is constrained by dose-dependent cardiotoxicity. Pegylated liposomal doxorubicin (PLD) has been proposed as an alternative.

**Material and method:**

This retrospective, registry-based cohort study investigates the efficacy of PLD in patients with locally advanced or metastatic STS treated at Aarhus University Hospital, Denmark, between 2008 and 2023. Patients were identified from a regional database, and progression-free survival (PFS) and overall survival (OS) were analysed.

**Results:**

A total of 38 patients were included, with 6 diagnosed with EHE and 16 with angiosarcoma. Among EHE patients, all had metastatic disease at diagnosis, with a median PFS of 7.8 months and OS of 1.5 years from the start of PLD treatment. Two patients remained progression-free for over 5 years. In angiosarcoma patients, the median PFS was 7.4 months, and the median OS was 2.4 years. Other STS subtype including solitary fibrous tumours (SFT), showed minimal benefit from PLD, with a median PFS of 2.8 months.

**Interpretation:**

Pegylated liposomal doxorubicin demonstrated clinically relevant activity in angiosarcoma and EHE. It may be considered a therapeutic option for patients with these aggressive vascular sarcomas. Further prospective studies are warranted to confirm its efficacy and optimised treatment strategies.

## Introduction

Locally advanced and metastatic soft tissue sarcoma (STS) accounts for 1% of all cancers and includes more than 80 distinct histopathological subtypes [1]. They are among the most challenging cancers to treat since they are heterogeneous in biological behaviour, resulting in varying sensitivity to treatment depending on the histological subtype. Furthermore, STS often lack a response to conventional chemotherapy [2, 3].

Epithelioid haemangioendothelioma (EHE) and angiosarcoma are subtypes of STS that arise from the endothelium and are often associated with blood vessels [4]. These two subtypes account for 2–3% of all sarcoma diagnoses [5].

The incidence of EHE is under 1/1.000.000 and is therefore characterised as a very rare cancer It originates from a blood vessel and is characteristic of WWTR1-CMTA1 breakpoint analysis [6, 7]. Epithelioid haemangioendothelioma has a very heterogeneous clinical presentation, as it can present in numerous primary sites, often in soft tissue, bone, liver, and lung. The disease is predominant in women, with a ratio of 4:1 [8, 9]. About 50% of the EHE patients present with metastatic disease at the time of diagnosis, primarily in the lung, liver, and bone [10]. This disease is diagnosed in patients of all ages; however, most cases are seen in young and middle-aged, with a median age of 36 years.

The clinical course of EHE can be indolent and have a slow progression [4]. However, EHE shows intermediate-grade malignancy as it still has the potential to recur or metastasise [4].

On the contrary, angiosarcoma are highly malignant, and there is a high prosperity of recurrence and metastasis, making it a very aggressive tumour [4]. The prevalence is more common in men than women; the peak age is the seventies [5]. Angiosarcoma presents in any site of the body, more commonly in soft tissue than in bone, with the scalp, neck, and face being the most predominant sites. In addition, radiation-induced tumours in the breast are often angiosarcomas [3–5].

Both EHE and angiosarcoma can pose a clinical dilemma based solely on their rarity and their limited responsiveness to most chemotherapy agents and, therefore, remain a challenging disease to treat. Especially young patients with EHE are dying from the disease, which is why it is essential to investigate effective treatments [2].

The current standard treatment for locally advanced or metastatic STS is doxorubicin, an anthracycline-based chemotherapy that remains the first-line option either as monotherapy or in combination regimes [11, 12]. Treatment choice depends on multiple factors, including tumour histology, patient performance status, age, and comorbidities [13]. However, the widespread use of doxorubicin has shown limited efficacy, with response rates generally below 40% [3]. In addition, its dose-dependent cardiotoxicity and other adverse effects often limit its long-term use, particularly in patients with pre-existing cardiovascular risk factors.

Given these limitations, alternative formulations such as pegylated liposomal doxorubicin (PLD) have been explored as potential treatment option for STS. Pegylated liposomal doxorubicin is a modified form of doxorubicin encapsulated in liposomes, which allows for prolonged circulation time, reduced reticuloendothelial system uptake, and improved tumour penetration. Pegylated liposomal doxorubicin has demonstrated efficacy in locally advanced and metastatic STS while offering a more favourable toxicity profile than conventional doxorubicin; however, the number of studies is low [14, 15]. A phase II trial of PLD versus doxorubicin treatment in 95 patients diagnosed with locally advanced and metastatic STSs, including 11 patients diagnosed with angiosarcomas, showed that PLD and doxorubicin had equivalent objective response rates (ORRs) and significantly better progression-free survival (PFS) in favour of PLD [15]. A smaller study, including 6 patients diagnosed with angiosarcomas treated with PLD, showed clinical benefit in 5 cases with only 1 patient with progressive disease (PD) within the first 6 months [3]. Another study investigated PLD treatment in 11 patients. One (EHE) patient had a progression-free interval of 60 months after withdrawal of PLD after seven cycles [16].

These data all support PLD activity; therefore, it has a potential role in treating local and metastatic STSs [3, 15–17].

The main aim of this study is to investigate the effects of PLD in locally advanced/metastatic STSs in a consecutive, retrospectively analysed cohort in a single sarcoma centre, primarily focussing on angiosarcomas and the very rare endothelial haemangioendothelioma.

## Material and methods

### Study design

This study is a registry-based cohort study of locally advanced/metastatic STS treated with PLD at the Department of Oncology, Aarhus University Hospital (AUH), Denmark, between the years 2008 and 2023.

### Patient cohort

The patients were identified from the Regional Database (BI-portal), which contains comprehensive information about all treatments given at the department.

Patients had to meet the inclusion criteria: (1) a diagnosis with locally advanced and/or metastatic STS, (2) age above 18 years, and (3) treated with PLD in the timeframe of 2008–2023. A total of 43 patients were included. Three patients were excluded as they had an allergic reaction to the treatment and did not complete the first treatment. Two patients had desmoid tumours and were excluded. This resulted in a cohort of 38 patients. To compare patients with EHE, angiosarcoma and solitary fibrous tumours (SFT) not treated with PLD with those treated with PLD patients from the Danish Sarcoma Database within the same time patient and with histological confirmed EHE, angiosarcoma and SFT were used as reference groups.

### Statistics

Our primary endpoint was PFS, from the date of the first PLD treatment until progression of the disease, defined as the start of another treatment or death, whatever comes first. The scans were not used to evaluate PFS, as some of the angiosarcomas were evaluated by clinical examination of the skin and some of the EHE patient progressed very slowly and the treatment was started at the time of clinical symptoms or based as shared decision with the patients. The secondary endpoint was overall survival (OS). Patients still alive on the last day of follow-up were censored. The data are presented as median survival time with a range (min and max). Data analysis was done using STATA statistical software version 18.1.

### Ethics

The study was approved by the Research Ethics Committee 1-10-72-2333-12 and the Internal Directory of Research Projects (journal number 1-16-02-741-18).

## Results

In all, 38 patients diagnosed with locally advanced- or metastatic STSs treated with PLD were included. The median age at diagnosis was 64 years (range 19–83 years). Table 1 shows the patient characteristics.

**Table 1:** Patients, tumour and treatment characteristic.

Characteristic	Total	Angiosarcoma	EHE	SFT	Other
		Number (%)	Number (%)	Number (%)	Number (%)
Sex					
Male	18	7 (44)	3 (50)	5 (56)	3 (43)
Female	20	9 (56)	3 (50)	4 (44)	4 (57)
Age					
Median years (min–max)	64 (19–83)	71 (55–83)	61 (52–71)	61 (36–70)	57 (19–74)
Tumour size (min–max)					
Median cm (min–max)	7 (0–35)	5 (0–12)	3.75 (2–4)	12.5 (5–27)	11 (6–35)
Tumour grade					
Low/borderline	3 (9)		1 (20)		2 (29)
Intermediate	8 (23)	3 (19)	2 (40)	3 (42)	
High	24 (69)	13 (81)	2 (40)	4 (57)	5 (71)
Primary stage					
Localised	25 (66)	12 (75)		7 (78)	6 (86)
Metastatic	13 (34)	4 (35)	6 (100)	2 (22)	1 (14)
Time from diagnosis to start PLD*					
Median months (min–max)	9.75 (0.1–163)	14 (1–88)	1 (0.1–8.4)	11 (1–118)	37 (1–162)
Lines of treatment before PLD					
Median (min–max)	0 (0–3)	0 (0–3)	0 (0–0)	0 (0–0)	1 (0–3)
Number of PLD cycles					
Median (min–max)	4 (1–10)	6 (1–10)	5 (4–6)	2 (2–5)	2 (1–6)
Lines of treatment after PLD					
Median (min–max)	4.5 (0–10)	4.5 (0–5)	0 (0–10)	5 (4–9)	3 (0–8)

EHE: epithelioid haemangioendothelioma; PLD: Pegylated liposomal doxorubicin; SFT: solitary fibrous tumours.

The median duration between the diagnosis and the beginning of PLD was 9.75 months (range 0.1–163 months)

Of the 38 patients, 6 were diagnosed with EHE, and none had prior treatment with chemotherapy. All were metastatic at the time of diagnosis. For these patients, the median time from the start of PLD until progression was 7.8 months (range: 3.7–76.3 months). At follow-up, 4 patients had died, and 2 were still alive. In general, the median OS from the time of diagnosis for this group was 1.5 years. Two patients, who were still alive, had not progressed after the end of PLD and had been followed for a median of 5.2 years. From the entire sarcoma cohort, only three patients with EHE did not receive PLD. These three patients had a median OS of 0.04 months.

**Figure 1:**
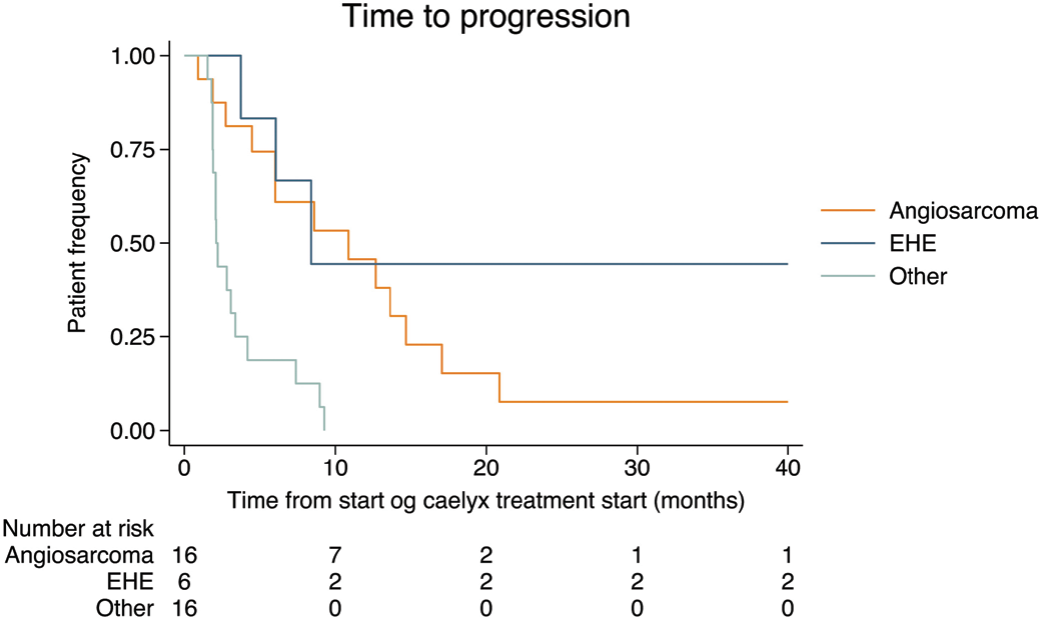
The time from the start of pegylated-liposomal doxorubicin treatment until progression or change of treatment for the different histological subtypes.

**Figure 2:**
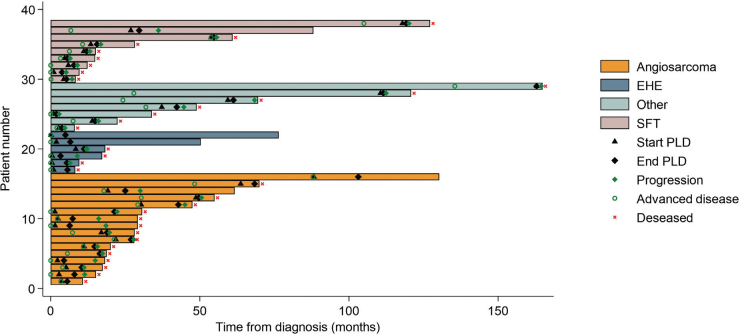
The swimmers’ plot shows the time from diagnosis until the start of pegylated liposomal doxorubicin (PLD), the end of PLD treatment, time to progression and patient status.

A total of 16 patients were diagnosed with angiosarcoma. Whil2 4 patients had metastasis at the time of diagnosis, 12 had localised disease. The median time from diagnosis until relapse was 14 months. Patients with metastatic or locally advanced angiosarcomas were treated with chemotherapy or radiotherapy, when surgery was not possible. Two patients received another type of chemotherapy before treatment with PLD. In all, 14 patients received PLD as the first line of treatment when locally advanced or metastatic disease occurred. The median time from treatment starting with PLD until progression defined as change to another treatment was 7.4 months (range: 0.9–42 months). At follow-up, 14 patients had died, and 2 were still alive. The median OS from the time of diagnosis was 2.4 years An additional 72 patients were registered with an angiosarcoma and were not treated with PLD in the time period; the median OS of these patients was 1.8 years. Some of the patient had localised disease and were treated with surgery, some patients did not receive PLD due to poor performance status or a reaction to PLD. The rest of the PLD-treated patients (N = 16) were diagnosed with other types of sarcomas, of which 9 were SFT. Four patients were treated with PLD due to previous excellent responses to doxorubicin, either as a complete response or stable disease, for more than 1 year. The histological diagnosis of these patients were one with unspecified sarcoma, one with synovial sarcoma, one with leiomyosarcoma, and one with retroperitoneal liposarcoma. Two patients were treated with PLD because of prior treatment with doxorubicin or epirubicin for another cancer; the histology of these patients were unspecified sarcoma and undifferentiated pleomorphic sarcoma. One patient with spindle cell sarcoma started doxorubicin with a good response but, after a few treatments, developed heart failure with decreased ejection fraction and was switched to PLD. At the time of diagnosis, two patients with other types of STS had metastatic disease. Five patients were treated with another form of chemotherapy before the use of PLD. The median time from starting treatment with PLD until progression defined as change to another treatment was 2.2 months (range: 1.5–9.3 months). For the SFT patients, the median time to progression was 2.8 months (range: 1.8–9.2 months) . The median OS for SFT patients treated with PLD was 2.6 years. In addition, 40 SFT patients were identified in the sarcoma database, and their median OS was 6.4 years. This additional cohort included both SFT with localised and metastatic disease. Figure 1 shows the time from PLD start until progression for patients with angiosarcoma, EHE, SFT, and other STSs. The time course for diagnosis for the individual patients is depicted in Figure 2.

**Figure 3:**
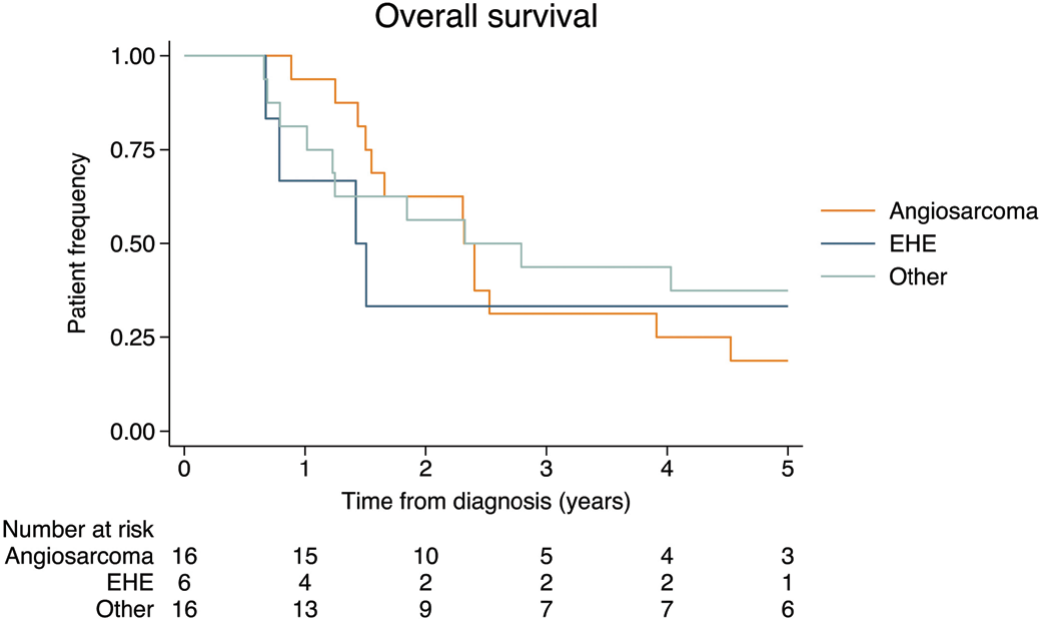
The overall survival of patients treated with pegylated-liposomal doxorubicin is timed from the start of diagnosis until death of any causes.

The OS of the patients from the time of diagnosis is shown in Figure 3. Overall survival at 2 years was 55% (95% Confidence Interval [CI]: 36–68) and at 5 years 29% (95%CI:15–43). For EHE, the OS at 2 years was 33% (95%CI: 5–68) and 33% (95%CI: 5–67) at 5 years. For angiosarcoma, the OS at 2 years was 63%(95%CI: 35–81) and at 5 years 19% (95%CI: 5–40).

## Discussion

The results show that PLD is a good treatment option for angiosarcoma and EHE with a median PFS of 7.4 and 7.8 months, respectively. For other sarcoma subtypes, such as SFT, it does not seem to have a clinical benefit.

Choosing the optimal therapy for patients with EHE remains challenging without good prospective clinical trials with a control arm. The slow pace of growth during treatment or the watchful wait for any treatment makes it hard to interpret reports of clinical benefit without adequate controls [4]. Some of the adverse effects of doxorubicin, which limits the treatment, are cardiomyopathy and myelosuppression. Furthermore, nausea, mucositis, and alopecia are adverse effects that are to be considered [12–14]. Studies have shown that treatment with PLD is less toxic for some adverse effects than doxorubicin. The cumulative PLD dose does not equal the increased cumulative myocardiopathy as seen with doxorubicin treatment, and PLD is significantly less myelosuppressive than doxorubicin.

In 2021, a consensus paper on the treatment of EHE was published. The primary treatment for unifocal EHE is surgery at an experienced sarcoma centre. When treating metastatic disease, chemotherapy has limited activity [18]. The recommendations from this consensus paper are, among others, sirolimus. This is based on two studies showing ORRs of 11 to 50%, and a medium PFS on 13–22 months [19, 20]. Another recommendation is pazopanib. It has shown an effect with a ORR of 20% and PFS of 26 months [21]. In all, 10 EHE patients were included in this study. The median OS of our EHE cohort was 18 months, and all were metastatic at the time of diagnosis; the fact that all patients had metastatic disease at the time of diagnosis could be due to selection bias, as the search in the database was done for a patient treated with PLD and not patient with EHE.

In the case of angiosarcoma, the rarity and the lack of randomised clinical trials to guide treatment is essential [4]. Angiosarcomas are sensitive to taxanes, which can be used as first-line treatment. Paclitaxel, given weekly as a single drug, has shown an ORR of 7% to 53% [22]. Treatment of angiosarcoma with paclitaxel has shown a PFS of 6.6 months and an OS of 19.5 months [23]. A more extensive randomised study compared doxorubicin versus paclitaxel for treating angiosarcoma. The ORR for doxorubicin was 29%, and for paclitaxel, 53%. The PFS for doxorubicin was 3 months versus 5.8 months for paclitaxel. The OS was 10.3 months for paclitaxel versus 5.5 months for doxorubicin [24]. Paclitaxel has been tested in combination with bevacizumab in angiosarcoma in a randomised phase 2 study. The combination therapy had the identical PFS as paclitaxel alone at 6.6 months [23]. Regarding the use of PLD, a randomised study including 94 angiosarcoma patients showed similar ORR and PFS doxorubicin; however, the treatment is less toxic [25]. A retrospective study of 125 patients with angiosarcoma found PFS of 4.2 months for treatment with PLD (11 patients had received this treatment), 4.0 months for paclitaxel (41 patients), 2.2 months for mono drug gemcitabine (11 patients), and 1.6 months for ifosfamide (12 patients) [26]. Pazopanib used in vascular sarcomas has shown ORR of 23%, DCR of 54%, PFS of 3 months, and OS of approximately 10 months [21]. Our results show a better effect of PLD with PFS at 7.4 months. One published case report has tested the combination of PLD with paclitaxel with a complete response [27]. This is a very interesting case. However, more cases need to be published to confirm that the combination is better than either of the drugs alone. Another game-changing therapy for many cancers is immunotherapy with checkpoint inhibitors. Nevertheless, most sarcoma patients do not respond to this treatment except for angiosarcoma [28, 29]. Checkpoint inhibitors is a promising new treatment, especially in combination with chemotherapy for angiosarcoma patients.

This population-based study includes all patients from one institution treated with PLD with 2 years of follow-up after starting PLD. One limitation of the study is the low number of patients treated, making it difficult to include all details. For example, the range of treatment after PLD for EHE patients was 0–10 with a median of 0. Only one patient was treated with 10 lines of treatment after PDL, and this patient had a 1.4-year OS. This study is retrospective in nature, and the low number of patients included in the different cohorts should be considered when interpreting the data.

## Conclusions

The effect of PLD in locally advanced and metastatic angiosarcoma and EHE have a clinically relevant effect and could be considered as a treatment option for these rare cancers.

## Data Availability

The datasets in this study are not publicly available. This is in accordance with the rules concerning processing personal data described in the European Union (EU) General Data Protection Regulation (GDPR) and the Danish Data Protection Act. However, should a researcher be interested in our data, they are welcome to contact the corresponding author.
